# Inclusion-light or innovation of inclusion: modes of innovation and exnovation for the German vocational rehabilitation and participation system

**DOI:** 10.3389/fresc.2024.1436003

**Published:** 2024-09-10

**Authors:** Jana York, Jan Jochmaring

**Affiliations:** Department of Rehabilitation Sciences, TU Dortmund University, Dortmund, Germany

**Keywords:** vocational rehabilitation, people with disabilities, inclusion, work, innovation, exnovation, participation

## Abstract

This paper examines the German system of vocational rehabilitation and participation from a system- and innovation-theoretical perspective. The German system of vocational rehabilitation and participation, with its established special systems for participation in the labor market, is facing a - long overdue - reorientation. The article presents central instruments of the vocational rehabilitation system based on legal foundations, official labor market statistics, and current research findings. The authors compare the legal requirements for an inclusive work environment with the actual employment situation of people with disabilities and highlight a central dilemma of inclusion. Two modes of innovation and exnovation in the vocational rehabilitation system are proposed and critically discussed to resolve the dilemma.

## 1 Introduction

Despite legislation, the German vocational rehabilitation and participation system is characterized by special systems, solutions and strategies and needs a reorientation. The chances of people with disabilities to participate in the general labor market are still lower than those of people without disabilities (1).

This theoretical article aims to make the German vocational rehabilitation and participation system accessible to an international readership and to facilitate future system comparisons. The article addresses three central questions:

1. What are the instruments of labor market policy that are used in the German system of vocational rehabilitation and participation?
2. What are the current opportunities for people with disabilities to participate in working life?
3. How can the existing system of vocational rehabilitation and participation be improved through exnovation or innovation?

To answer these questions, we analyze relevant national disability legislation, labor market statistics, and research findings using document analysis. We discuss our findings in terms of system and innovation theory.

First of all, we outline our understanding of impairment and disability and of German social legislation. The International Classification of Functioning, Disability and Health (ICF) (2) and the German participation report (3) distinguish between impairments and disabilities. People with impairments are defined as those who have damage to bodily structures or functions or mental disorders that permanently impair their performance in activities related to these impairments (3). A disability is no longer seen as a characteristic of a person, but rather as the result of a problematic interaction between individual prerequisites and environmental conditions or contextual factors (3). In this understanding, we assume that a person is not disabled, but rather becomes disabled due to conditions in the labor market. German social legislation, which is relevant to this article, works with the constructs of severe disability to address benefits for participation in the workforce, categorizing people as able to work, able to work in a workshop and unable to work. Depending on the context, the terms people with impairments, disabilities or severe disabilities are used here.

The second section “General labor market” focuses on the central laws governing the participation of people with disabilities in the workforce, as well as the labor policy control mechanisms aimed at promoting their employment in the general labor market.

The third section “System of vocational rehabilitation and participation” describes the central instruments of the vocational rehabilitation system, including their legal basis, objectives, and degree of dissemination. A distinction is made between instruments that focus on participation in a special labor market or participation in the general labor market.

The fourth section titled “Participation in working life between ambition and reality” analyzes the employment rates of people with and without disabilities, the vocational transition processes, and the structure of the instruments of vocational rehabilitation against the background of the political-normative inclusion claim of the Federal Republic of Germany.

The fifth section “Innovation and exnovation in the vocational rehabilitation and participation system” outlines two modes for innovating the vocational rehabilitation system: Current-Inconsistent-Mode and New-Consistent-Mode. The Current-Inconsistent-Mode describes the current constitution of the vocational rehabilitation system, where traditional special systems continue to exist alongside newly introduced instruments. This article explores strategies for expanding inclusion-oriented instruments that have already been introduced and dismantling special systems. The New-Consistent-Mode proposes an exnovation of all sheltered systems and raises practical implementation and ethical questions from a system-theoretical perspective. It then presents strategies for developing new impulses for participation in a dialogical way through co-creation and participatory research.

In the concluding discussion (section six), the modes undergo a critical reality check.

## 2 General labor market

Altered work realities challenge traditional logics of welfare state action and delineate innovation needs. Processes of digitization, the pluralization of employment relationships, as well as educational expansion – to name just a few examples – generate novel opportunities as well as risks for the participation of people with disabilities in the workforce (1). Skill shortages and changing demands on employment relationships and times challenge employers. The pressure to innovate in labor markets also affects the employment opportunities of people with disabilities (4).

In the Federal Republic of Germany, there exists a broad legislative framework for promoting the participation of people with disabilities in the workforce: the Federal Participation Act (Bundesteilhabegesetz), the General Equal Treatment Act (Allgemeines Gleichbehandlungsgesetz), the Disability Equality Act (Gesetz zur Gleichstellung von Menschen mit Behinderungen), and the Law on the Promotion of an Inclusive Labor Market (Gesetz zur Förderung eines inklusiven Arbeitsmarktes), which was announced in June 2023. With the ratification of the United Nations Convention on the Rights of Persons with Disabilities by the Federal Republic of Germany in 2008, a normative-political inclusion postulate was elevated to a key objective of socio-political action (5). Article 27 on work and employment proclaims: “States Parties recognize the right of persons with disabilities to work, on an equal basis with others; this includes the right to the opportunity to gain a living by work freely chosen or accepted in a labor market and work environment that is open, inclusive and accessible to persons with disabilities” (5). Since then, Germany has been committed to the guarantee and promotion of this right.

The above-mentioned laws require employers to contribute to the participation of people with disabilities in the workforce by training, hiring or retaining. A central legal instrument for participation in working life is the employment obligation of people with disabilities and the associated compensation levy. According to § 154 of the Ninth Social Code (Neuntes Sozialgesetzbuch), private and public employers with an average of at least 20 employees per month are required to contribute through employment or compensation levy to integrate severely disabled people into work. If employers do not fulfill the employment obligation, they pay a so-called compensation levy, which is staggered depending on the degree of fulfillment of the employment obligation (§ 160 Ninth Social Code). An analysis of the fulfilment of the obligation to employ severely disabled persons for the reporting year 2021 reveals that only 39% of all employers fully met their mandatory obligations, 35% partially met them, and 26% did not meet them (6).

In addition, Occupational Integration Management (Betriebliches Eingliederungsmanagement) is an instrument designed to help individuals who acquire an impairment during their working life to remain employed. It is an instrument designed to enable employees who are ill or at risk of disability to remain in or return to work. Employers are obliged to offer Occupational Integration Management if employees are unfit for work for more than six weeks without interruption or repeatedly within a year (§ 167 Ninth Social Code). Currently, only about 40% of eligible employees receive an offer for Occupational Integration Management. This percentage is even lower in smaller companies, in skilled trades businesses, and the service sector (7, 8).

The vocational rehabilitation and participation system offers a range of active labor market policy instruments to promote the participation of people with disabilities in working life, within the legal framework mentioned earlier. However, the existing instruments are designed differently and differ in their scope, number of users and socio-political orientation from a participation perspective. The following section explains the central instruments of the vocational rehabilitation system.

## 3 System of vocational rehabilitation and participation

What are the instruments used by the system of vocational rehabilitation and participation? The German system of vocational rehabilitation and participation offers a wide range of instruments for the realization of participation in working life for people with disabilities. These instruments of vocational rehabilitation are divided by the authors into instruments aimed at participation in a special or in the general labor market. Special instruments generate subsystems that are largely decoupled from the general labor market. Access to these instruments is controlled by social policy via a definition of people with disabilities who are entitled to access. Vocational rehabilitation instruments that are designated for the general labor market assist employment relationships that are subject to social insurance contributions outside of specific systems (see Table 1).

**Table 1:** Instruments for participation in working life.

Instruments special labor market	Instruments general labor market
- Special Needs Day Care Centers *(Heilpädagogische Tagesförderstätten)* - Sheltered Workshops for People with Disabilities *(Werkstatt für behinderte Menschen)*	- Inclusive Companies *(Inklusionsunternehmen)* - Supported Employment *(Unterstützte Beschäftigung)* - Budget for Work/Training *(Budget für Arbeit/Ausbildung)* - Support in working life *(Begleitende Hilfen im Arbeitsleben)* - Work Assistance *(Arbeitsassistenz)*

There is a broad special labor market with Special Needs Day Care Centers and Sheltered Workshops for People with Disabilities, which are intended to prepare people with disabilities for participation in working life. Workshops for people with disabilities enable participation in the special labor market, but in an exclusionary system. At the same time, they make qualified employment in the general labor market impossible (1, 9). Inclusive Companies, Supported Employment and the Budget for Work or Training open up opportunities for participation in the general labor market with their support services, but the potential for inclusion remains limited by definition and accessibility. The instruments support in working life and Work Assistance generate or stabilize employment in the general labor market.

In the following, the instruments for labor participation in the vocational rehabilitation system are explained in terms of their legal basis, objectives and degree of dissemination.

### 3.1 Instruments special labor market

#### 3.1.1 Special Needs Day Care Centers

Paragraph 219 of the Ninth Book of the Social Code outlines provisions for the care and support of people with disabilities who are unable to perform a minimum amount of economically viable work. These individuals may attend Special Needs Day Care Centers, which may be affiliated with a Sheltered Workshop for People with Disabilities. The primary aim of these centers is to prepare individuals for participation in working life. According to (10), just over 38,000 people attended Special Needs Day Care Centers nationwide in 2020.

#### 3.1.2 Sheltered Workshop for People with Disabilities

As per Paragraph 219, Section 1 of the Ninth Social Code, workshops are facilities that allow people with disabilities to participate in working life. These workshops provide vocational training or employment opportunities for individuals who are unable to work on the general labor market due to the nature or severity of their disability (Ninth Social Code § 219). However, only individuals who can provide a minimum level of economically viable work performance are eligible for admission to the workshop (Ninth Social Code § 219). Workshops are also legally obligated to facilitate the transition from the workshop to the general labor market. Outsourced jobs, also known as company-integrated or external jobs, are offered on the first labor market for the purpose of transition. Their number is not systematically recorded in statistics (11). Currently, there are approximately 320,000 people working in Sheltered Workshops for People with Disabilities, including those in external jobs and the vocational training sector (10, 12).

### 3.2 Instruments general labor market

#### 3.2.1 Inclusive Companies

According to Paragraph 215 of the Ninth Social Code, Inclusive Companies are legally and economically independent companies or public employers in the general labor market. In addition to their regular economic activities, Inclusive Companies have a social mission of employing, training, and placing people with disabilities. Inclusion Companies hire individuals with severe disabilities for the general labor market. These individuals may face particular difficulties in finding employment due to the nature or severity of their disability or other circumstances, despite having exhausted all support options and the use of integration specialists (§215 Ninth Social Code). Inclusive Companies employ 30 to 50% of individuals with severe disabilities and offer employment subject to social insurance contributions with collectively agreed or customary local pay. According to Bundesarbeitsgemeinschaft Inklusionsfirmen (13), 13,590 individuals with severe disabilities were employed in 975 Inclusive Companies.

#### 3.2.2 Supported Employment

The objective of Supported Employment is to enable people with disabilities to obtain or retain appropriate and suitable employment subject to social security contributions (§ 55 Ninth Social Code). It consists of two components: individual in-company training and vocational support (§ 55 Ninth Social Code). Individual in-company training involves a trial of appropriate in-company activities, preparation for an employment relationship subject to social insurance contributions, and induction and training at an in-company workplace. Occupational support services aim to stabilize an employment relationship subject to social insurance contributions. Approximately 3,000 people with disabilities use Supported Employment (14). However, it is assumed that there is a significantly greater potential for Supported Employment (15, 16).

#### 3.2.3 Budget for Work/Training

People with disabilities who are entitled to benefits in the work area of a Sheltered Workshop for People with Disabilities receive the Budget for Work if they enter into an employment relationship subject to social insurance contributions (§ 61 Ninth Social Code). The benefits also provide guidance and support in the workplace, in addition to a wage cost subsidy to compensate for the reduced performance of the employee (§ 61 Ninth Social Code). In 2020, a total of 1,679 Work Budgets were paid out, including all cases from 2018 (10).

People with disabilities who are entitled to benefits during the initial process or vocational training in a sheltered workshop for people with disabilities receive the training budget when they sign a training contract with a private or public employer (§ 61a Ninth Social Code). The training allowance is reimbursed, including the employer's share of the total social security contribution and the accident insurance contribution. Additionally, expenses for necessary guidance and support at the training place and vocational school, as well as travel costs, will be covered. The Budget for Training covers additional costs incurred by completing the school-based part of the training outside of a regular vocational school in a vocational rehabilitation facility. It is provided until the successful completion of the training. As of October 2023, only 62 Budgets for Training have been granted nationwide (17). Therefore, it plays a minor role in funding statistics.

#### 3.2.4 Support in working life

The Integration Office, in collaboration with the Federal Employment Agency and rehabilitation providers, offers support in working life to prevent a decline in the social status of severely disabled individuals. The aim is to provide employment opportunities that allow them to utilize and develop their skills and knowledge, and to compete with non-disabled individuals in the workplace (§ 185 Ninth Social Code). In addition to providing financial assistance to people with disabilities and employers, the accompanying support also includes psychosocial assistance from specialist integration services (§185 Ninth Social Code). As part of the accompanying assistance, people with severe disabilities are, for example, entitled to financial benefits for technical aids, to establish and maintain an independent professional existence and to participate in measures to maintain and expand professional knowledge and skills (18). Support in the workplace includes advisory services and financial benefits for employers, such as creating disability-friendly work and training opportunities for people with severe disabilities (18). Currently, there is a lack of adjusted statistics that allow statements about the overall volume and usage numbers of this instrument.

#### 3.2.5 Work Assistance

The purpose of Work Assistance is to enable people with severe disabilities to participate in working life if they need assistance in carrying out their work but are otherwise able to fulfil their contractual obligations (18). According to (19), employees with severe disabilities are responsible for organizing and instructing their assistants. They can either hire them themselves (employer model) or commission an assistance provider to aid assistance at their own expense (service model). In 2020, 3,577 people received work assistance services from the integration offices (18).

## 4 Participation in working life between ambition and reality

What is the situation regarding the participation of people with disabilities in the German workforce? To answer this question, the employment rates of people with and without disabilities are first compared and transitions from school to training and from training to employment are analyzed. Next, the development of special systems and more inclusion-oriented structures will be analyzed, based on the degree of dissemination of vocational rehabilitation instruments. Finally, this section discusses whether the current opportunities for labor participation align with the political and normative demands of inclusion, or if there are any missed ambitions.

### 4.1 Employment rate and transition

In 2021, there were three million severely disabled people aged 15 to under 65. The employment rate of people with severe disabilities in this age group who were subject to social insurance contributions was 47.8%, which is significantly lower than the rate of 75.6% for people without disabilities (6). Although the employment rate of people with disabilities has increased (2005: 41.6%) in line with the general increase in employment (6), this increase is mainly due to internal company recruitment of employees who have acquired a disability during their working life (1, 20). However, access to training or employment in the general labor market has not substantially improved (21).

Disadvantages are particularly evident in the area of transitions from school to training and from training to employment. According to Blanck (22), Jochmaring (21), and Zölls-Kaser (23), the transition from special systems to the general labor market is also more difficult. Despite a legally enshrined transition mandate, the transition rate from a Sheltered Workshop for People with Disabilities to the regular labor market is currently only 0.1–0.2% (24, 25).

### 4.2 Working realities between workshops for people with disabilities and inclusion-oriented instruments

In recent decades, the largest special system, Sheltered Workshop for People with Disabilities, has steadily expanded. The number of employees has doubled in the last 20 years to around 320,000 (10, 12, 26). Currently, employment in workshops is stagnating at a high level (10). Workshops absorb people with disabilities from the general labor market by keeping them in segregating systems. They enable participation in labor, but not in the general labor market (9).

This development is linked to the fact that although there are inclusion-oriented instruments available, the number of users is too low. Currently, only around 3,000 people work with Supported Employment in the general labor market (14), which represents a significant imbalance compared to the 320,000 people currently employed in Sheltered Workshops for People with Disabilities (12). A comparison of the number of users of the sheltered workshop instrument, with currently 320,000 employees, and the Budget for Work instrument, with currently 1,700 employees, also shows significant differences. At present, significantly more people with disabilities are employed in the special workshop system than in the general labor market with the Supported Employment or Budget for Work instruments (10, 14, 27).

### 4.3 Resolving the inclusion dilemma?

In summary, it can be stated that the goal of creating an inclusive world of work is currently not being achieved. Special systems, solutions, and paths are in direct opposition to the ideals of the Convention on the Rights of Persons with Disabilities (5). There is no automatic process that translates political-normative inclusion claims into real opportunities for participation in the workforce.

We discuss two ways to address this inclusion dilemma: by deviating from the objective of creating an inclusive work environment or through a change in the German system of vocational rehabilitation and participation in line with the objectives of the legislation. The following outlines the first way for change in appropriate brevity. The fourth section, “Innovation and exnovation in the vocational rehabilitation and participation system”, discusses the second way in detail.

Frings (28) proposes establishing participation as a vision for society as a whole, rather than anchoring inclusion as a socio-political objective. According to Frings (28), setting the “goal of participation” instead of the programmatic “goal of inclusion” can break the currently prevailing cycle of problem solving and problem creation. The current welfare state intervention logic can only be broken by turning away from the ideal state of inclusion. At present, the welfare state is faced with tasks that are essentially aimed at recognizing disability as a problem that needs to be solved, making conditions and circumstances related to disability public, and developing (legally) binding solutions to problems that guarantee inclusion in various subsystems of society. From a system-theoretical perspective, the goal of participation can facilitate the inclusion of specific functional systems and the creation of individual solutions that are specific to those systems, in addition to opening up new discourse arenas (28). This expands the view beyond the institutionalized differentiation between “inside” and “outside” and considers varying spaces of possibility on different social levels.

## 5 Innovation and exnovation in the vocational rehabilitation and participation system

We first explain the underlying understanding of innovation and exnovation, before describing two modes of innovation and exnovation for the German vocational rehabilitation and participation system.

Innovations in the vocational rehabilitation system can be both technical and social in nature. The focus here is on social innovation as a response to key societal challenges that cannot be solved by technical innovation alone (29). Howaldt and Schwarz (30) define social innovations as intentional and targeted reconfigurations of social practices within a specific field of action, such as vocational rehabilitation. The objective of social innovations is to enhance problem-solving capabilities compared to established practices. Unlike technical innovations, social innovations are not only analyzable but can also be brought about through reflexive processes (30). Applied to the vocational rehabilitation system, this refers to social practices that create more opportunities for participation in the world of work. Exnovation is defined as the dismantling and abolition of systems and social practices (31–33). This article explicates those systems and social practices as worthy of exnovation, in light of the reference foil of inclusion. This implies that they are no longer expedient or even harmful for inclusion.

As a theory-led “thought experiment”, the authors propose two modes of innovation and ex-innovation for the vocational rehabilitation system:

1. The Current-Inconsistent-Mode, and
2. The New-Consistent-Mode.

Both modes aim to explore how opportunities for participation can be expanded and barriers removed. They mark discourse arenas that are not without overlaps, are even more interdependent and in some cases can only release innovation or exnovation potential if they are closely interlinked.

Inspired by the “onion model” (34), the two modes are described in terms of their legal, structural and behavioral logics (see Table 2).

**Table 2:** Innovation and exnovation logics.

	Legal logic	Structural logic	Action logic
Current-Inconsistent-Mode	Guiding through a confusing system	Path dependencies – realities of allocation, funding, and counselling Scaling inclusion-oriented instruments	Recruitment practices Institutionalized pathways to work
New-Consistent-Mode	Reduction of complexity	System conservation and reconfiguration	Co-creation and participatory research Self-determined choices

### 5.1 Current-Inconsistent-Mode

This mode describes the current situation of the vocational rehabilitation system in Germany: in addition to newly introduced instruments such as Supported Employment, which aim to enable participation in the general labor market, the dominant special systems, in particular the Sheltered Workshops for People with Disabilities, remain in place. This situation is considered inconsistent as it remains undecided due to its focus on socio-political control: The “inclusion-light version” moves between targeted change and the preservation of established systems. In this mode, inclusion-oriented instruments are introduced in small amounts, while special systems remain largely untouched.

The paper discusses ways to reduce barriers to participation and increase opportunities in an inconsistent mode (see Table 2). The following topics are analyzed: (1) Guiding through a Confusing System, (2) Path Dependencies: Realities of Allocation, Funding, and Counselling, (3) Scaling Inclusion-oriented Instruments (4) Recruitment Practices and (5) Institutionalized Path-ways to Work.

#### 5.1.1 Guiding through a confusing system

The complexity of the vocational rehabilitation system poses a problem for creating more opportunities for participation in the general labor market. The legal structure of existing vocational rehabilitation instruments is too complex, and their practical application is too complicated to be easily communicated or utilized. For example, the complexity of the system means that it is difficult for responsible actors to provide advice because they do not have an overview of the wealth of instruments, some instruments are too unfamiliar, and structures for transitions between responsible institutions need to be developed (35, 36). This can make it challenging to access inclusion-oriented services easily.

To address the complexity of the vocational rehabilitation system, counselling structures have been created to appeal to both employers and people with disabilities. Since January 2022, inclusion offices have been tasked with setting up Standardized Contact Points for Employers (Einheitliche Ansprechstellen für Arbeitgeber – EAA) nationwide (§ 185a Ninth Social Code). The purpose of Standardized Contact Points for Employers is to increase opportunities for people with severe disabilities and those with equivalent status by proactively advising and addressing employers. These Standardized Contact Points for Employers are intended to have a “guide function” in the vocational rehabilitation system (§ 185a Ninth Social Code). The organization provides low-threshold information, advice, and support to employers regarding the training, recruitment, and continued employment of people with disabilities. They also establish contact with service providers, such as the Federal Employment Agency or Pension Insurance providers (37). Furthermore, the Supplementary Complementary Independent Participation Counselling (Ergänzende Unabhängige Teilhabeberatung – EUTB) (§ 32 Ninth Social Code) is a service that provides individuals with disabilities with information and guidance on rehabilitation and participation benefits. The counselling service complements the existing counselling structures of the service providers and offers advice and guidance (38).

#### 5.1.2 Path dependencies – realities of allocation, funding, and counselling

The current allocation of people with disabilities to special systems is hindering their ability to participate in the general labor market. The issue lies in the ease of entry into the special system, without adequate or accessible exit routes. While legal exit routes exist, they are not practical in use. We are specifically dealing with a permanent extension of the exclusionary institution Sheltered Workshops for People with Disabilities. This topic has been subject to scientific criticism (39, 40) and has also been denounced by self-advocacy organizations of people with disabilities (41, 42).

Furthermore, the current limitations in funding and counselling hinder the expansion of vocational rehabilitation programs that promote inclusion. The advice provided by the Federal Employment Agency is based on the number of places “purchased” in the previous year, and financial resources are allocated for future “purchases” on this basis (15, 43). Current funding mechanisms result in ongoing mediation within the existing system, which has been identified as requiring change (44).

According to Blanck (22) and Rosenberger (45), the counselling situation focuses less on openly exploring career opportunities and more on assigning individuals to established structures. Consequently, there is less emphasis on counselling for employment options that are perceived as insecure, such as Supported Employment (27, 46). Referring individuals to exclusion management institutions creates a barrier to participation in the general labor market (9). The aforementioned challenges can be overcome by implementing new referral mechanisms and by consistently exploring new employment opportunities.

#### 5.1.3 Scaling inclusion-oriented instruments

The scaling of existing instruments appears to be beneficial for increasing opportunities for participation in the general labor market.

Outsourced workplaces in workshops have the potential to create employment relationships subject to social insurance contributions on the general labor market. This is especially true for younger, able-bodied people who have not been working in a workshop for long (47). These jobs, integrated within the company, can serve as a “hinge function” that allows both employees and companies to experience labor under normal conditions (47). However, outsourced workplaces also carry the risk of hindering transitions to the general labor market, as they offer benefits to people with disabilities, employers and the workshop system compared to regular employment. Individuals with disabilities typically earn higher wages in outsourced employment settings compared to sheltered workshops, and they also receive retirement benefits. Employers may enter into favorable contracts with workshops, resulting in economic benefits for both parties. Additionally, workshops can benefit from increased profits and a better reputation (48, 49).

The participatory and empowering structures of the Supported Employment instrument align with the goal of creating an inclusive world of work. Evaluations of the instrument confirm its effectiveness in enabling participation in the general labor market and increasing opportunities for long-term employment (14, 50). Possible changes to broaden the instrument include simplified access, expanding the target groups, and considering new forms of employment and support formats (51). To simplify access to Supported Employment, especially for in-company training, quotas could be increased. Additionally, vocational guidance should be reformed. Currently, only individuals with a recognized severe disability are eligible to participate in vocational guidance (14). This creates a paradoxical situation where more than half of Supported Employment recipients cannot receive long-term support within the framework of vocational guidance after completing in-company training. Research findings suggest that continuous vocational support increases the chances of a long-term employment relationship (52). The instrument could reach additional target groups, particularly people with mental illness who do not have a recognized severe disability, by removing barriers to access. In designing the Supported Employment instrument, it is important to consider the growing flexibility of labor and company organization (53). This could include the possibility of supported self-employment or the creation of supported mobile service groups.

Similar to the Supported Employment instrument, the Budget for Work/Training instrument has the potential to create employment relationships subject to social insurance contributions on the general labor market. To increase the participation of people with disabilities in work through the Budget for Work/Training instrument, it needs to be structurally simplified. To make the application process more manageable, especially for people with learning difficulties, it is necessary to reduce bureaucracy. This can be achieved by considering scientific evidence on existing barriers and criticism from organizations representing those affected (27, 46).

Even in the case of Occupational Integration Management, which is a targeted support instrument for staying employed, the number of offers and users indicates that legal requirements have not been fully implemented (54). According to a representative survey of employees, only about 40% of those who are potentially entitled have received an offer for Occupational Integration Management. Of these, just under 70% have accepted the offer (7, 8). Larger companies more frequently provide Occupational Integration Management than smaller ones (54). It is particularly prevalent in companies where Health Management is already established and where there is an appreciative and supportive management culture (55).

The current advisory structures, including the Standardized Contact Points for Employers (37) and the Supplementary Complementary Independent Participation Counselling (38), can aid in raising awareness of person-centered services, such as Work Assistance or support in the workplace, and removing barriers to access.

#### 5.1.4 Recruitment practices

Research on transition and vocational training has shown that people with disabilities face limited opportunities to enter the general labor market (21). Employers also create recruitment barriers (56–58). The extent to which matching or fit problems in companies are responsible for the lack of employment opportunities for people with disabilities is questionable (56–58). Companies often justify their decision not to hire or employ people with disabilities on the grounds of their inadequate level of performance, based on existing societal performance standards and expectations (58–60). Critics often argue that there are no available job openings or that the necessary qualifications are not met (58–60). Other arguments include references to the high level of bureaucracy or the lack of knowledge about support services (56, 61–63).

#### 5.1.5 Institutionalized pathways to work

The professional careers of individuals with disabilities, particularly those with learning difficulties, are closely associated with the workshop system. Vocational assignment processes, as compared to career choice processes, have an even greater impact (45). When leaving the sheltered workshop system, people with disabilities are confronted with the hardships of a capitalist labor market, including the possibility of dismissal, probationary periods and high-performance requirements, as well as precarious employment relationships. In contrast, the workshop system provides concrete and achievable opportunities for work that are similar to regular employment, as well as security in retirement (25). For example, workshop employees receive compensation for disadvantages in the form of a pension top-up: under pension law, they are assessed as if they earned 80% of the social security reference amount (64). These tendencies in the workshop safety space make leaving the system unappealing and risky in the medium and long term. Typically, re-entry into the Sheltered Workshop for People with Disabilities from the general labor market requires a new application and is subject to availability of places (21).

#### 5.1.6 Interim conclusion

In order to innovate the system of vocational rehabilitation and participation in an inconsistent manner, opportunities for participation in the general labor market must be gradually expanded. Simultaneously, access to existing special systems must be made more challenging. The article describes possible control mechanisms for increasing opportunities for participation in the general labor market in the future. These mechanisms are related to the allocation processes in special systems, the control of financing and advice realities, and scalability potentials that have been previously explained. Currently, professional career opportunities in specialized systems, employer recruitment practices, and the complexity of the vocational rehabilitation system hinder these opportunities.

### 5.2 New-Consistent-Mode

The New-Consistent-Mode refers to the thorough removal of exclusive systems and structures in vocational rehabilitation. The de-institutionalization of special systems, which contradict the socio-political primate of inclusive work environments, breaks away from the previous organizational logic in vocational rehabilitation through irritation. This mode aims to challenge the explicit expansion and reproduction logics of segregating systems by structurally provoking change scenarios. The disruption of the established system of vocational rehabilitation and participation is “radical”: It raises questions about the whereabouts of those people with disabilities employed in special systems, the ethical reasonableness of de-institutionalization and new socio-political solution scenarios.

In addition, the removal of exclusive systems creates a supply vacuum. Human and economic resources that were previously allocated to special systems could be reallocated through participatory, dialogue-based negotiation processes, such as co-creation or participatory research. Innovation impulses can arise from new perspectives of all stakeholders involved in the vocational rehabilitation system. Participatory dialogue or the empowering development of new instruments to increase opportunities for participation in working life are conceivable approaches to bringing something new into the world (65). Co-creation and participatory research are two approaches that aim to promote collaboration and participation of various stakeholders in innovation processes.

These topics are the subject of further discussion below: (1) Reduction of system complexity, (2) System conservation and reconfiguration, (3) Co-Creations and participatory research and (4) Self-determined choices (see Table 2).

#### 5.2.1 Reduction of system complexity

As mentioned earlier, the vocational rehabilitation system is so complex that advisory structures have been created to help employers and people with disabilities navigate the system. Various organizations, including the Federal Employment Agency, Statutory Pension, Health and Accident Insurance, and Integration Services, offer services to support labor participation. However, the multitude of contact persons and instruments can cause confusion for those seeking support, support providers, and decision-makers (28).

In order to facilitate greater participation in the general labor market, the complexity (66, 67) of the vocational rehabilitation system would have to be significantly reduced. Potential avenues for innovation include the bundling of responsibilities and a reduction in the number of contact persons. The question of which actor is solely responsible for which vocational rehabilitation topic would have to be negotiated.

In addition to the definition of responsibility, this also includes the process of defining the profile and thus also clearly identifiable selectivity of the individual actors/stakeholders and decision-makers. At the same time, this ensures connectivity. Following the central ideas of Luhmann's system(s) theory (67), these are essential characteristics for decisions in organizations (68). Another way to reduce complexity would be to reduce or merge laws and regulations - which would then facilitate decision-making and connectivity (67, 68).

#### 5.2.2 System conservation and reconfiguration

According to the central findings of Luhmann's sociological systems theory (66, 69), organizations and institutions tend towards self-preservation. With the autopoietic turn, in which Luhmann (66, 69) emancipated himself from Parsons (70, 71) and Maturana and Varela (72), he assumed that systems reproduce themselves out of themselves, or rather maintain themselves. If we start from this mechanism of reproduction and apply these theoretical insights to the specific system of Sheltered Workshops for People with Disabilities, it becomes clear that the workshops cannot abolish or change themselves, but will always look for ways to reproduce themselves.

From a systems-theoretical perspective, sheltered workshops are currently doing exactly what they were designed to do: They provide target group specific integration into work. In other words, they literally provide integration services, but they do not achieve inclusion, contrary to the political-normative postulate of inclusion (21). Without an “irritation” of the existing system, no “reconfiguration” of the system is conceivable, at least in theory (28).

#### 5.2.3 Co-Creations and Participatory Research

Co-creations and participatory research differ in their objectives and application processes. Co-creation aims to develop new products, services or solutions together with different stakeholders by bringing in their specific knowledge, skills and perspectives. Participatory research enables community members or stakeholders to be involved in research activities, ensuring their voices, needs and perspectives are considered. Co-Creation specifically focuses on the collaborative development of services or products, whereas participants in participatory research are involved in the entire process (question formulation, data collection, intervention, interpretation).

From the perspective of innovation theory, traditional methods of social action can be reconfigured through new forms of cooperation, known as co-creations (30). In the context of vocational rehabilitation, co-creations could generate innovative impulses for participation in the workforce. Co-created products, such as instruments for increasing opportunities for participation in the general labor market, arise from the understanding that complex tasks are best addressed when all those affected by a specific problem can participate equally and with their individual knowledge, skills and perspectives in the development of solutions. This approach creates more sustainable results (73). Co-creation extends beyond co-production (73) and can mitigate paternalistic power structures. It is not a specific method of working, but rather a strategy that enables actors to collaboratively develop and plan something new (74). Eckhardt and Krüger (73) define co-creation as a basic mindset and holistic philosophy, which includes joint exploration of a consensus on the specific subject area and ways to change it as part of the innovation process.

Innovations to open up opportunities for participation may also arise from new insights gained through participatory research. “Participatory research approaches are rooted in social movements that stand up for a democratic and inclusive society” (75). In the sense of the International Collaboration for Participatory Health Research (76), participatory research is to be understood as a research paradigm. The central aim is “to maximize the participation of those whose life or work is the subject of the research in all stages of the research process” (76). Furthermore, participatory research is locally situated and collectively owned with the aim of achieving a positive social transformation of the realities of the lives of the people involved. The knowledge generated in the research process is local, collective, co-created, dialogical and diverse (76). Participatory research enables the reflection of power structures in a dialectical process and aims to have a broad impact (76).

Both strategies facilitate the active involvement of individuals with disabilities in the creation of more inclusive work environments. Through co-creation, individuals with disabilities can become innovators themselves and play a pivotal role in shaping their own working reality. Within participatory research and development approaches, the focus is on the needs of individuals with disabilities. Furthermore, they are empowered to transition from a role of aid recipient to that of an autonomous creator of their own working reality.

#### 5.2.4 Self-determined choices

For people with disabilities, the new mode offers opportunities to shape their own professional biographies. The abolition of special systems opens up new ways into the world of work and opportunities for self-determined career choices.

On the one hand, this is a gain and can contribute to the expansion of professional and financial autonomy. On the other hand, this gain in freedom is also associated with risks (77). The consequence is that one's own professional biography has to be shaped and constantly reshaped (78). The ambivalence lies in the fact that the gain in freedom can simultaneously create new dependencies and risks, and that the individual is increasingly responsible for success or failure in his or her professional life (77).

Opportunities also lie in the changing world of work as a result of increasing technologization, digitalization and automation, as well as the pluralization of forms of employment that can be observed. These changes can open up new occupational segments for people with disabilities (1).

#### 5.2.5 Interim conclusion

The rigorous de-institutionalization of special systems opens up new perspectives for inclusion. However, it also means an arrangement with the performance pressure of capitalist modes of production (79). Inclusion in the educational or economic system of capitalist societies offers opportunities for participation but also carries new risks of exclusion. Inclusion involves confrontation with expectations of normality as well as with the market-driven selections and impositions of the labor market (1, 80). However, the comparison with better-performing employees, although conveying “normality”, also results in poorer (employment) opportunities due to lower performance (80).

From the perspective of innovation theory and a participatory research paradigm, this text describes two ways to develop, establish, and scale new ideas for the vocational rehabilitation system in a co-creative and participatory manner. Goals can also be negotiated from multiple perspectives by establishing new types of discourse. In this way, it would be possible to critically reflect on whether the abolition of special systems is actually the desired goal of all those involved or whether special paths should or must be retained.

## 6 Discussion

Currently, social policy action in Germany is inconsistent and unable to resolve the tension between claims of inclusion and the reality of inclusion. The existing system of vocational rehabilitation and participation alone cannot make the necessary structural changes for inclusion. The long-term sustainability of changes in “homeopathic doses” and the acceptance of a “participation light” by politics and society remain uncertain.

The New-Consistent-Mode require socio-political clarification regarding the situation of people who are exposed to the (performance) demands and hardships of a capitalistically organized work system when special systems are dismantled. Currently, there is a lack of widely supported political visions, designs, and practical concepts.

Special systems like the Sheltered Workshops for People with Disabilities continue to fulfil important central tasks. They provide daily structure and concrete practical implementation of participation in working life, leading to labor market integration, but not inclusion. Rehabilitation-specific programs in vocational preparation and training also create a bridge to employment opportunities, even if they are less demanding (21).

Discussing the compatibility of workshop expansion dynamics with the provisions of the Convention on the Rights of Persons with Disabilities (5), York et al. (4) suggest that current dynamics represent a structural and organizational problem rather than a solution. Under certain socio-political conditions, the continued and increased absorption of people with disabilities from the general labor market requires explanation (1). As stated by Hüppe (81), this may be contrary to fundamental rights, or as summarized by Sackarendt and Scheibner (82), contrary to human rights. Politically, there is disagreement on how to deal with the expanded workshop system. This is not surprising given that political decision-makers are facing powerful opponents in the form of the organizations that run the workshops. The German organizations responsible for Sheltered Workshops for People with Disabilities have become the largest group owners in the service sector for people with disabilities in Europe (81).

If one were to take the claim of inclusion and its consequences seriously and argue in favor of dismantling special systems, this would also mean having to provide employment policy perspectives for a growing group of people. This poses a central challenge to the established functioning of capitalist labor societies and their fixation on performance and skills (83, 84). The issue at hand is the level of tolerance for underperformance or “dis-ability” that is accepted in a capitalist economic system (85).

Political action that intervenes to regulate, but does not want to touch, capitalist principles of performance reveals a dilemma: on the one hand, more inclusion should be achieved and special systems should be abolished; on the other hand, the capitalist ethos of performance should not be touched (21). Moreover, there is no plan to resolve this contradiction and to exnovate the special systems.

The two innovation and exnovation modes, understood as a theory-led “thought experiment”, enable the construction of different scenarios, which will be further differentiated in subsequent publications and supported with empirical findings. It would be beneficial to conduct international comparative research on the management of specialized systems in vocational rehabilitation and participation in the future. The German system, which is described in this paper, could be contrasted with other systems to gain insight into several key questions. These include: How do other countries implement the Convention on the Rights of Persons with Disabilities (5) in the context of work? What challenges are encountered? And what insights can be derived from the experiences of other countries?

## Data Availability

The original contributions presented in the study are included in the article, further inquiries can be directed to the corresponding author.
